# Conceptualizing and Treating Comorbid Chronic Pain and PTSD

**DOI:** 10.1155/2013/174728

**Published:** 2013-06-02

**Authors:** Michelle A. Bosco, Jessica L. Gallinati, Michael E. Clark

**Affiliations:** James A. Haley Veterans Affairs Hospital, University of South Florida, 13000 Bruce B. Downs Boulevard (116B), Tampa, FL 33612, USA

## Abstract

The purpose of this paper is to review the rationale for concurrent, evidence-based treatment of chronic pain and posttraumatic stress disorder (PTSD). To meet this end, we review pertinent definitions and extant theories related to the two conditions and their correlations with each other. We then synthesize theoretical components into a proposal of a comprehensive conceptual framework for understanding the relationship and clinical complexity of overlapping chronic pain and PTSD. We conclude with an example of an integrated treatment model designed specifically to address a fundamental factor associated with pain and PTSD: avoidance.

## 1. Introduction

The purpose of this paper is to provide sound rationale and clinical recommendations for the concurrent, evidence-based treatment of chronic pain and posttraumatic stress disorder (PTSD). After reviewing current definitions and conceptualizations of both chronic pain and PTSD, the paper elaborates and extends the fear-avoidance model of pain and PTSD to provide a comprehensive explanatory framework for the conceptual, symptomatic, and behavioral overlap of the two conditions. These clinical similarities, coupled with a review of common factors and shared mechanisms evident in the two conditions, are highlighted and are fundamental to the rationale for application of concurrent, evidence-based treatment of co-occurring symptoms or disorders associated with chronic pain and PTSD. Current programs or models applying concurrent treatment for veterans and service members returning from the ongoing conflicts in the Middle East (Operations Enduring and Iraqi Freedom, OEF/OIF; Operation New Dawn, OND) are identified, and preliminary outcomes regarding this approach's usefulness and effectiveness are summarized. Given the shared psychological and behavioral mechanisms, it may be argued that concurrent treatment of chronic pain and PTSD underway in the veteran population also would be beneficial to nonmilitary/nonveteran populations.

## 2. Prevalence and Impact of Chronic Pain

Turk [1] defines pain as a multidimensional, complex, subjective, perceptual phenomenon, a definition shared by many pain researchers. Chronic pain has been defined in the literature as pain lasting for greater than 3 to 6 months, persisting beyond the healing of the initial injury or disease process [2]. As the chronicity of pain increases, emotional distress, functional limitations, and increased utilization of the healthcare system tend to occur [3–6]. Chronic pain may develop gradually over time, seemingly independent of any known trauma, or it may develop in response to a specific injury. The cycle of chronic pain is self-perpetuating and self-reinforcing, as painful sensations lead to a tendency to guard and/or avoid activity or therapy, which in turn leads to atrophied muscles, decreased range of motion or flexibility, and ultimately serves to maintain the pain cycle.

The estimated prevalence of chronic pain in the general US population ranges from 10 to 20% [7]. One recent study [3] investigating the prevalence of pain, PTSD, and postconcussive syndrome in OEF/OIF veterans (*N* = 340) found that 10.3% of veterans evaluated reported the presence of *only *chronic pain after factoring out overlapping symptoms associated with chronic pain, PTSD, and persistent postconcussive syndrome (PPCS). The investigators' appreciation for this symptom overlap is consistent with other recent clinical research conducted by Gironda and colleagues [8] and with two studies conducted later by Clark and colleagues [9, 10] within the VA system of care. The impact of chronic pain on the community healthcare system has been well documented (see [4, 5] for reviews).

Chronic pain impacts the individual in various domains of functioning [4, 5]. Specifically, chronic pain may affect emotional functioning, resulting in depression, irritability, or anxiety. The ability to cope with pain may be compromised, resulting in the development of maladaptive or avoidant strategies to manage its effects. This ineffective coping likely reflects maladaptive cognitions held by individuals with chronic pain, consisting of attentional biases, memory biases, and depleted beliefs that one can effect change in one's life [11]. Relationships, occupational functioning, and social functioning may suffer greatly, and as the fear of pain (misinterpreted as ongoing damage) begins to dictate physical activities, physical functioning declines with an increase in sedentary and inactive behaviors.

## 3. Prevalence and Impact of PTSD

Posttraumatic stress disorder is defined by the revised fourth edition of the Diagnostic and Statistical Manual of Mental Disorders [12] as the development of a constellation of symptoms after experiencing or witnessing an extreme traumatic event. The emotional reaction to the event is characterized by horror, intense fear, or helplessness. Specifically, three symptom clusters develop and persist for at least one month in response to the traumatic event(s). Each constellation of symptoms requires that a number of criteria be met in order to meet the diagnosis for PTSD. First, at least one reexperiencing symptom must be reported. Second, at least three symptoms demonstrating persistent avoidance of trauma reminders and numbing of general responsiveness must be present. Third, at least two hyperarousal symptoms must be noted. These symptoms lead to clinically significant distress and/or impairment in social, occupational, or other functional domains. Should these symptom clusters persist beyond three months, the disorder is considered chronic in nature. The symptoms of PTSD may be conceptualized as an interactive cycle, in which reexperiencing phenomena lead to or become associated with feelings of increased physiologic arousal, which in turn results in a tendency to avoid trauma reminders or triggers in efforts to regulate the noxious physiologic hyperarousal. However, attempting to control reexperiencing symptoms and hyperarousal symptoms of PTSD by avoidance may actually worsen or intensify the experience of the event [13], perpetuating the cycle just described.

Prevalence rates for PTSD in the general population have been reported at 6% (males) and 12% (females; [7]). The prevalence of the disorder in the veteran population is theatre-specific: Vietnam-era veterans have exhibited a lifetime prevalence rate of 30.9% (men) and 26.9% (female; [14]) while Gulf War point prevalence rates of 10% for men and women have been reported [15]. Among OEF/OIF veterans, point prevalence rates were estimated at 68.2% of one sample in a large northeastern VA hospital [3], regardless of co-occurring symptomology. When PTSD was examined in isolation, it was found to reflect 2.9% of that percentage, but when chronic pain and PTSD were considered together specifically, the prevalence in Lew et al.'s OEF/OIF sample was 16.5%.

The impact of PTSD on functioning has been well documented. The disorder or its symptoms may lead to difficulties in emotional functioning, such as irritability, sadness, depression, guilt, shame, or anger. Psychiatric comorbidities are more common than not [16], and depression in particular has been found to frequently cooccur with chronic pain and PTSD [17]. Similarly, the energy to manage daily tasks is likely depleted with underregulated PTSD. To “regulate” the discomfort inherent in hyperarousal and the distress inherent in reexperiencing phenomena, an individual may develop chronic avoidant behaviors that act to prolong the disorder rather than foster improvement [18]. Social and interpersonal functioning likely are impacted in response to isolation prompted by avoidance and hyperarousal symptoms. For example, isolative behaviors that result from an attempt to regulate physiologic distress when faced with trauma reminders may lead to social withdrawal and decreased quality of relationships and less emotional, social, or familial engagement. Quality of sleep may diminish in response to reexperiencing phenomena such as nightmares or hyperarousal that limits the ability to sufficiently calm or relax the body to promote sleep. Occupational functioning may suffer as sleep, mood, and interpersonal domains decline. Physical health problems have been shown to be more common among individuals with PTSD, particularly in veteran populations [16, 19], and chronic pain has been reported to be one of the most frequent co-occurring physical problems for those with PTSD. Specifically, individuals with PTSD more often report the presence of chronic pain [20] while studies also show an increased prevalence of PTSD in chronic pain patients [21]. Additionally, individuals with chronic pain oftentimes report more severe PTSD symptoms [7] and when chronic pain results from a traumatic event, the prevalence of PTSD increases [6].

## 4. Prevalence and Impact of Co-Occurring Chronic Pain and PTSD

Associations between chronic pain and mental health disorders in general, along with growing evidence of the frequent co-occurrence of chronic pain and PTSD, have been documented in the literature [7, 10, 21–23], as has the elevated prevalence of PTSD in both civilian [22] and veteran chronic pain populations in the United States [3, 6, 16, 24]. For United States service members returning from OEF/OIF/OND, pain continues to be one of the most frequently reported symptoms [8, 25]. Injuries sustained on the battlefield in the current military actions may have been fatal in prior conflicts; however, advances in medical care far forward in the combat theatre combined with advances in protective armor have led to increased survival rates [26]. The frequent use of improvised explosive devices (IEDs), landmines, and rocket propelled grenades (RPGs) by insurgent groups in Iraq and Afghanistan highlights the importance of understanding the impact of repeated blast exposure on the human brain, body, and emotions. Nonblast injuries resulting from gunshot wounds, shrapnel, vehicular crashes, falls, and other physical traumas also contribute to the emotional traumas of war. Many OIF/OEF/OND returnees present with pain, persistent postconcussive syndromes, and PTSD or posttraumatic symptoms (PTS) with associated functional difficulties or deficits [3, 9, 10]. Lew and colleagues [3] report 42.1% of OEF/OIF veterans evaluated at a large northeastern VA medical center presented with a combination of overlapping pain, PTSD, and PPCS. While we acknowledge and appreciate the influence of chronic pain and PTSD on cognitive functioning, the scope of the present paper is the dyad of comorbid chronic pain and PTSD, its conceptualization, and the need for concurrent treatment models. The functional impact of co-occurring pain and PTSD conditions should not be ignored. Individuals with comorbid pain and PTSD report health problems with greater frequency [19, 27], along with more pain-related disability, higher pain ratings, and increased functional impairment. Additionally, they demonstrate higher rates of health care service utilization and increased health care costs. Occupational functioning is also at risk, as those with comorbid pain and PTSD demonstrate more frequent absenteeism [27] and greater loss of productivity [28].

## 5. Theoretical Considerations: Conceptualizing Co-Occurring Chronic Pain and PTSD

### 5.1. Chronic Pain

Given the multidimensional impact and experience of chronic pain, a useful conceptualization of chronic pain is that of the complex interaction of somatosensory, psychological, and social factors consistent with the *biopsychosocial model* [4, 5, 29]. This framework accounts for its fundamental complexities and fosters a range of clinically relevant treatment strategies to target pain-related dysfunction. Because pain is influenced by complex, interacting psychological/emotional, behavioral, physical, and sociocultural processes, a wide range of interventions targeting different aspects or components of the pain experience have been established. Pain beliefs develop over a lifetime of learning, reflecting both primary (degree of threat) and secondary (ability to control or cope with pain) meanings or appraisals associated with pain [30]. Pain beliefs and appraisals influence an individual's adjustment to the effects of pain in addition to that individual's emotional and behavioral responses to pain. Fear-avoidance models of chronic pain recognize the salience of this maladaptive belief process in addition to the emotional, fear-based component inherent in the models [31]. The beliefs that *chronic* pain is a signal of damage or harm and that activity should be avoided to recover from pain are widespread [32] and erroneous. The development of these beliefs with the associated avoidance behaviors may impede the natural, pain-related recovery from injury or disease process. The chronicity of fear-based cognitive structures, as described in Foa and Kozak's [33, 34] *emotional processing theory of PTSD,* appears to be a factor in the development of adaptive versus maladaptive avoidant responses to *pain *as well. Immediate, acute fear would be adaptive in maintaining an individual's safety and bodily integrity; however, as avoidance becomes chronic, over the long term, it interferes with recovery, rehabilitation, and overall quality of life.

### 5.2. PTSD

The *emotional processing theory* by Foa and Kozak [33, 34] provides a useful conceptual framework to understand PTSD, providing the foundation upon which Prolonged Exposure Therapy (PE) is based. The hallmark of emotional processing theory is the role of fear as cognitive structure, which includes representations of feared stimuli, fear response(s), and meanings associated with feared stimuli and fear responses. Fear structures may be adaptive when they represent actual danger and the actions needed to assure safety in response to that danger. However, fear structures may be maladaptive or pathological when they do not accurately reflect the environment, when fear or escape responses are provoked by harmless stimuli, when fear responses interfere with adaptive behaviors, or when harmless stimuli become erroneously associated with danger or threat. PTSD represents an interruption in normal recovery, as many who experience traumatic events develop PTS. However, these symptoms dissipate in a reasonable amount of time [18], as natural recovery occurs following adequate emotional processing and confrontation of objectively safe but feared trauma reminders. As such, PTSD is conceptualized as a pathological process that develops when emotional processing is interrupted or curtailed secondary to chronic avoidance, avoidance that is further fueled by the development of maladaptive beliefs of the self, others, and the environment in general.

### 5.3. Synthesizing Conceptualizations for Chronic Pain and PTSD

The *fear-avoidance models* of chronic pain [35] and PTSD [18, 36, 37] noted above stress interacting concepts of feared stimuli, maladaptive beliefs or misinterpretations of physiologic arousal, and resultant avoidance behaviors ultimately maintained by negative reinforcement to explain each condition. Chronic pain and PTSD share the clinical features of fear and avoidance, which may influence the development of each condition over time, may serve to maintain them, and may interact in ways that impact the outcome of either condition [6, 24]. Fundamentally, feared stimuli (both pain and trauma-related) are chronically avoided, leading to physical, emotional, and cognitive repercussions that impede recovery. In short, fear-based avoidance appears to be a salient common denominator for the development and maintenance of chronic pain and PTSD as demonstrated separately in each specialty's own body of literature. Nevertheless, conceptual bridges and nescient empirical research focusing specifically on the processes underlying a *shared relationship* between the two are underway and reviewed below.

The *mutual maintenance model* (MMM) [38] suggests that shared factors may maintain both chronic pain and PTSD. This model posits that attentional biases, or a learned tendency to focus on certain internal and external stimuli, result in greater attention to threatening or painful stimuli. In addition, those with chronic pain and PTSD may demonstrate an increased sensitivity to anxiety's physiologic symptoms and tend to catastrophize, or expect the worst, to a greater extent than those without either of the two conditions. Identification of the physiologic sensation of pain as a trauma reminder and pain as a (mis)perceived indication of physical damage may trigger the comorbid disorders shared proclivity toward avoidance as a primary coping strategy. Depletion of cognitive resources, fatigue, lethargy, and general anxiety may result from shared factors and may contribute to psychological or physiological inertia or discomfort, catastrophizing tendencies, and subsequent avoidant coping.

The *shared vulnerability model* [20] is closely related to the MMM in that it shares its interoceptive focus, but it goes on to propose that higher anxiety sensitivity (i.e., sensitivity to physiologic signs of anxiety, such as pounding heart or shortness of breath) *predisposes* an individual to respond with greater fear in terms of emotional phenomenological experience and behavioral reactions to fear than do those with lower anxiety sensitivity. The tendency to respond with fear to physical symptoms of anxiety is deemed a shared vulnerability to the development of chronic pain or PTSD.

Despite models attempting to explain the conceptual relationship(s) between chronic pain and PTSD, the underlying mechanisms remain underinvestigated, prompting Leidl and colleagues [39] to attempt clarification of the mechanisms that may contribute to that relationship. The researchers conducted a one-year longitudinal study in Australia, measuring pain and PTSD at one week later injury/trauma, 3 months later injury/trauma, and 12 months later injury/trauma. The researchers found acute pain and pain at 12-month followup to be mediated by arousal symptoms at the 3-month assessment interval. They also reported significant pain-related mediation of acute and 12-month follow-up assessment of reexperiencing phenomena and arousal symptoms, concluding these findings as evidence of a mutual maintenance-type model of pain and PTSD. As noted above, this literature is in its infancy, and we encourage the expansion of this area of investigation through studies designed with strong methodological integrity.

**Figure 1:**
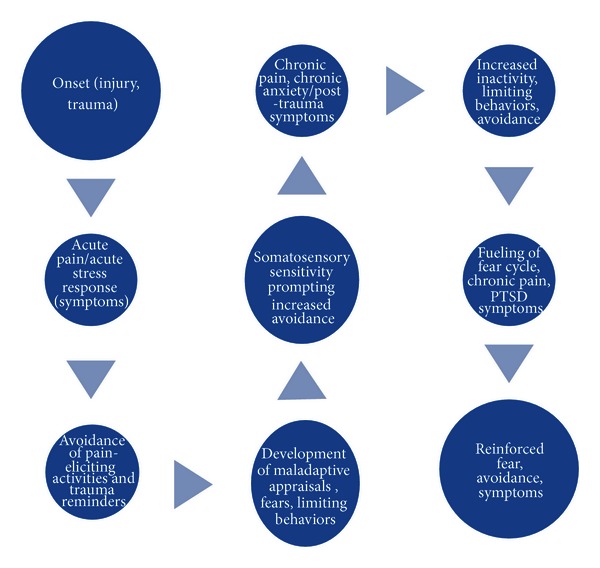
Comprehensive fear-avoidance cycle of chronic pain and PTSD.

While chronic pain and PTSD may share similar psychopathological sequelae in response to chronic fear and avoidance behaviors, the precise etiology and specific clinical expression of these processes may differ. Nevertheless, it is proposed that a salient common denominator maintaining both chronic pain and PTSD is chronic avoidance, reflecting fundamental fears to each condition and essentially reflecting a failure to recover and rehabilitate from physical and/or psychological injury (see Figure 1). For example, fear of reinjury [40] may prevent an individual from engaging in appropriate activities or therapies to address a chronic pain condition. Additionally, expecting or believing only the worst may happen (catastrophizing) complicates the ability to manage chronic pain (see [5] for a discussion), as it may also interfere with engagement in appropriate activities or therapies. Furthermore, hyperawareness of bodily sensations may contribute to fears and avoidance behaviors. Similarly, in PTSD, fear of (objectively safe) trauma reminders may prevent an individual from engaging in previously enjoyed activities as well as from confronting anxiety-provoking stimuli. Additionally, because the anxiety-provoking stimuli are chronically avoided, a maladaptive expectation or belief develops (e.g., “I cannot handle this”), which further complicates effective engagement in behaviors that would facilitate the natural recovery process. In essence, individuals with chronic pain and/or PTSD allow themselves to be controlled by pain and/or distressing symptoms.

As outlined in Figure 1, our integration of fear-avoidance models and mutual maintenance/shared vulnerability models into a comprehensive framework emphasizes the *cyclical* nature of fear and avoidance in the development and maintenance of co-occurring chronic pain and trauma-related symptoms and functional difficulties. We propose that within the acute phase of both chronic pain and PTSD, the initial injury or trauma sets into motion a cascade of adaptive responses or expected symptomology that, over time, become maladaptive, actually serving to maintain the condition rather than correcting it. Specifically, immediately following an injury or traumatic event (which may be one and the same), removing oneself from the danger prevents further tissue damage/pain or traumatization, as safety is of utmost importance. Short-term avoidance, then, is an adaptive, expected response to acute injury or trauma. However, chronic avoidance represents a maladaptive process that serves to maintain both PTSD and the functional limitations associated with chronic pain.

In sum, the fear-avoidance cycles associated with both chronic pain and PTSD are self-perpetuating, resting on a common foundation of avoidance that precludes recovery and reinforces maladaptive beliefs, ineffective behaviors, distressing symptoms, and functional limitations. We focus on the fear-avoidance construct within this cycle, as we see this factor as essential to the maintenance of the other behaviors and symptoms associated with both chronic pain and PTSD. We do not dispute that other shared factors reflect similarities between the two conditions, but we conceptualize their maintenance and functional implications within the context of a cycle of chronic avoidance.

## 6. Practical Considerations: Treating Co-Occurring Chronic Pain and PTSD

The remainder of this paper focuses on the practical or behavioral role fear and avoidance play in maintaining chronic pain and PTSD, and how confronting fears inherent in each disorder is a fundamental component to concurrent treatment. Concurrent treatment relies heavily on evidence-based therapies (EBT) or interventions that have been shown effective for treating chronic pain and PTSD and that essentially serve to deflect or minimize the shared role of fear-avoidance. First, we review cognitive behavioral theory and the rationale for EBTs to address these conditions. Next, we review integrated treatment components designed for concurrent treatment prior to introducing programs that incorporate them. 

Cognitive behavioral theory (CBT) proposes that thoughts affect how one feels and behaves. This relationship is dynamic and multidirectional. Cognitions or thoughts develop in part as a result of social learning [41] and may be adaptive or maladaptive in scope. The role of reinforcement, or the strengthening of behavioral responses, and the influence that consequences of actions or behaviors have on intensifying or maintaining a given level of functioning are important concepts influencing CBT. The degree of accuracy or inaccuracy of cognitions is a critical factor in CBT, as it underlies its definition of psychopathology. Specifically, CBT conceptualizes psychopathology as maladaptive cognitions that influence and interfere with emotions and behavior. Maladaptive cognitions with their associated affective states may prevent behavioral change necessary to successfully address both chronic pain conditions and PTSD.

Specific to chronic pain, treatment may focus on actively managing physical symptoms, maladaptive beliefs, and emotional symptoms or more broadly on the processes that contribute to the development or maintenance of the pain cycle. Interdisciplinary pain programs (IPPs) are particularly effective in altering these processes, especially for those with complex pain conditions or moderate to severe pain-related disability [4, 5, 42]. Treatment approaches targeting maladaptive processes in one fashion or another have been shown to be useful in addressing the complexity of the pain cycle [4]. Core IPP treatment approaches include CBT with components structured to challenge maladaptive pain beliefs and appraisals through psychoeducational sessions. Additionally, relaxation training is taught to counteract the effects physical tension has on pain levels. Physical therapy comprised of active modalities is an important component of IPPs to increase strength, flexibility, and confidence. These components are similar to the cognitive-behavioral approaches to PTSD treatment in terms of behavioral change, challenges to maladaptive beliefs (directly or indirectly), and behavioral activation so important to reengaging in enjoyable activities. By targeting cognitions directly and through behavioral tasks or strategies that promote confidence and a sense of agency, CBT facilitates a shift to more adaptive cognitions, emotional processing or experience, and behaviors necessary for recovery or rehabilitation from both chronic pain and PTSD.

Empirically supported treatments for both PTSD and chronic pain oftentimes stem from CBT concepts or interventions. We propose that an integrated treatment approach must rely on EBTs for each condition, and to this end we integrate Otis and colleagues' [24] and Foa et al.'s [18] treatment suggestions together while incorporating elements of IPP. The result is an integrated, multidisciplinary behavioral health program in operation at a large, southeastern VA hospital that provides services to veterans suffering from both trauma and chronic pain. Underlying this approach is the philosophy that treating comorbid chronic pain and PTSD must begin with a thorough assessment of each condition [6], including a comprehensive clinical, diagnostic interview; administration of outcomes measures at specified, repeated intervals during treatment; and application of empirically supported cognitive-behavioral interventions either individually or as part of a multidisciplinary treatment approach. As shared mechanisms of fear and avoidance maintain the impairments associated with chronic pain and PTSD, confrontation or exposure to feared stimuli is likely a fundamental ingredient for successful management and treatment of both chronic pain and PTSD. Concurrent treatment of pain and PTSD should target shared fear-avoidance processes and associated functional deficiencies. 

As avoidance maintains the disability or dysfunction associated with pain and PTSD, it may be argued that repeated confrontation or exposure-based interventions should facilitate engagement in the behaviors and activities that would allow for behavioral change, cognitive restructuring, and emotional growth. Consistent with Foa and colleagues' [18] rationale for conducting exposure-based interventions for PTSD, two conditions must occur to modify fear structures: (1) activation of the fear structure itself and (2) the fear structure must be challenged with contradictory or incompatible (i.e., corrective) information. Deliberate, systematic confrontation with objectively safe stimuli provides the impetus to modify the fear structures and alter the pathological cognitions that have developed about the self and the world. Similarly, as activities that have been avoided due to chronic pain are systematically approached and confronted, the body gains strength and flexibility and self-efficacy increases: rehabilitation for chronic pain may be argued to activate fear structures associated with cognitions related to pain and is likewise challenged by incompatible information. As this process unfolds, the individual also learns to tolerate negative affect, physiologic signs of anxiety/fear, through the habituation process. As feared activities are attempted repeatedly, they begin to lose their potency. 

Treatment goals for both chronic pain and PTSD sufferers focus on improvement in daily functioning and increasing quality of life by decreasing avoidance behaviors. Shared treatment goals for a treatment protocol or program concurrently treating pain and PTSD are outlined presently and represent an elaboration of common treatment mechanisms first proposed by Otis and colleagues [6]. See Table 1 for a summary of the treatment components described in this section.

First, psychoeducation about the interaction between behavior, emotion, and cognition reflecting the development of ascribing meaning, or appraisals, related to both chronic pain and PTSD is a key component to psychological interventions for both pain management (see [5] for a review) and the treatment of PTSD [18]. Particular attention should be paid to the propensity to catastrophize, as this thought process negatively influences pain rehabilitation outcomes [43]. Providing education about the interaction between physical, emotional, behavioral, and cognitive spheres is important in addressing pain and PTSD concurrently, as each condition interacts with and influences the other [6, 24]. Consistent with IPP approaches, education about the impact of pain and emotional status on cognitive functions such as attention, concentration, and memory would also be of benefit in the concurrent treatment of comorbid pain and PTSD. 

Second, a hierarchy of avoided stimuli should be constructed to serve as a basis for assigning situational exposure/confrontation exercises. This treatment element addresses the role of fear-avoidance in the development and maintenance of both chronic pain and PTSD. Similar to Foa and colleagues' [18] explanation of the in vivo “real life” hierarchy, patients may be asked to identify and rate on an agreed upon scale feared and avoided stimuli (including, e.g., behaviors, activities, and people) associated with chronic pain and PTSD or anxiety. The patient works systematically up the scale to more challenging items, building on a foundation of successes and increasing self-esteem. 

Third, interventions to reduce depression should be initiated. Depression has been suggested to be a potential mediator between pain and PTSD [17], and the co-occurrence of depression and chronic pain and depression and PTSD has been established in the literature (see [5, 17] for reviews). CBT interventions that have been shown to be effective in chronic pain and PTSD treatment efforts typically include a strong component of behavioral activation to facilitate reengagement in previously enjoyed activities, which tend to decline in the presence of depressive symptoms or a depressive disorder. These specific activation activities can be included in the pain/PTSD hierarchy described above.

Fourth, cognitive restructuring, either directly through thought logs with associated exercises to challenge maladaptive automatic thoughts or indirectly through the process of strengthening esteem and sense of agency/efficacy and worth via behavioral assignments and habituation, should be included in the concurrent treatment of pain and PTSD. Cognitive restructuring allows for maladaptive or distorted cognitions to be challenged with contradictory evidence and disconfirmatory experiences.

Fifth, correcting attentional biases that develop in response to both chronic pain and PTSD should be addressed in a concurrent treatment program or protocol. Individuals with chronic pain may overattend to potentially pain-inducing stimuli, whereas individuals with PTSD or PTS may overattend to potentially threatening stimuli. These biases may generalize to anything that may be perceived to cause or increase pain or be perceived as threatening. What Foa et al. [18] term “safety behaviors” serve as actions that protect an individual from the discomfort associated with anxiety-provoking stimuli. Education about the biases that may develop can be helpful for individuals with both chronic pain and PTSD or PTS. Identifying and ceasing safety behaviors associated with both pain and PTSD may prove beneficial if changes or reductions in distress are not forthcoming. 

Sixth, it is critical that emotional response and contributing physiologic sensations are normalized as part of treatment. Hypersensitivity or hyperawareness of physiologic sensations may be debilitating in their misinterpretations or overinterpretations. Normalizing emotional responses, providing education about the fight or flight response, and systematically addressing avoidance or escape behaviors are crucial to treatment success.

Structured relaxation training, which may include breathing exercises, is one method that can be used to help regulate physiologic symptoms of anxiety. Relaxation also assists in relieving tension, which may reduce pain levels. However, one potential contraindication for relaxation training for PTSD should be considered. According to Foa and colleagues [18], relaxation could be used to reduce emotional engagement in specialized PTSD trauma-focused work. If this occurs, it should be addressed and corrected.

Outcomes measures that assess chronic pain, PTSD, cognitive functioning, coping behaviors, and specific functional domains are necessary in assessing current functioning and levels of distress in those with chronic pain and PTSD to identify their emotional and behavioral sequelae and to assess their response to concurrent treatment. Initial assessment should include a thorough pain and mental health interview to inform treatment planning and provide outcomes data for programmatic growth and change. Intermittent measures should be administered throughout program participation to inform patient progress and treatment planning to address ongoing challenges. Communication and collaboration between providers, clinics, and specialties may enhance patient care when treatment is delivered by several independent providers rather than within multidisciplinary programs, which have the luxury of addressing concurrent, overlapping chronic pain and emotional difficulties in a targeted program format. Improving communication between providers and coordinating service delivery address problems in a more cohesive manner and prevent duplication of services.

## 7. Concurrent Chronic Pain and PTSD Treatment Options

**Figure 2:**
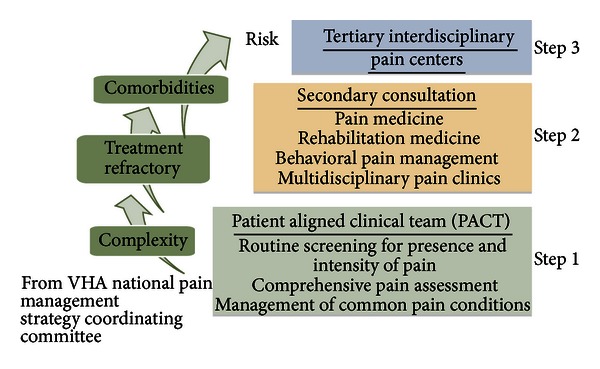
Veterans health administration stepped pain care model [45].

The VA System of Care has implemented a stepped approach to chronic pain management and rehabilitation [44, 45]. The first tier involves managing pain in the primary care setting with the inclusion (or proposed inclusion) of pain psychology embedded into the ambulatory pain setting for the purposes of assessment, time-limited treatment, or referral to other stepped care tiers. The second tier involves more specialized pain services such as interventional medicine, specialty pain clinics, or integrated care programs that treat pain and pain-related comorbidities concurrently or may involve referral to ancillary specialty programs that target coexisting disorders such as PTSD separately. The third tier encompasses interdisciplinary chronic pain programs that are comprehensive in their scope, philosophy, and approach to chronic pain rehabilitation and emphasize the importance of opioid cessation.  Figure 2 summarizes the basics of the VHA's stepped care model.

**Figure 3:**
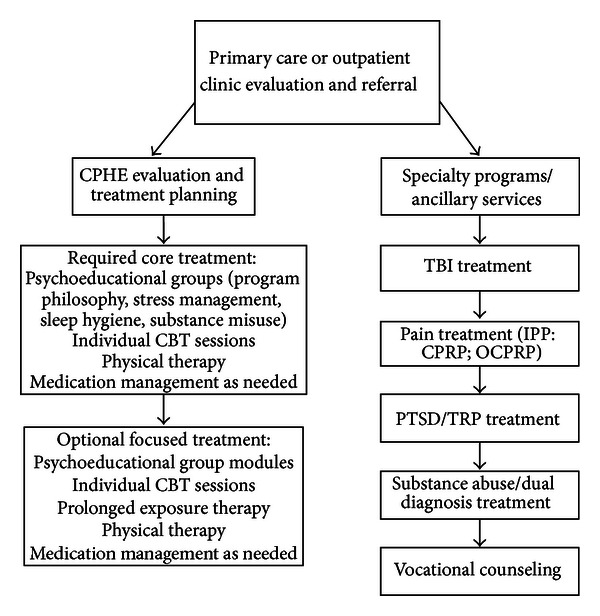
CPHE treatment components and relationship to ancillary services.

In this model, comorbid, integrated treatment of pain and PTSD occurs in step 2. One example of such a program is the Center for Postdeployment Health and Education (CPHE) located at a large VA facility in the US southeast. The CPHE is a time-limited, multidisciplinary program developed to treat concurrent, overlapping symptoms of pain, PTSD (or its symptoms), and mild cognitive complaints. The treatment protocol involves the six treatment goals mentioned earlier in this section, in addition to a seventh goal focusing on treating PTSD specifically with PE. Participants in the program engage in physical therapy, psychoeducational groups, individual psychological therapy, and psychiatry sessions on a regular basis (see Figure 3 for a summary of major program components). 

Outcomes measures are administered at intake, upon completion of the core portion of the program, and upon completion of any additional focused treatment components. Outcomes to date demonstrate broad improvement across the range of symptomatic measures collected including those specific to chronic pain and PTSD. During CPHE treatment, veterans or service members may also receive additional concurrent physical health or mental health treatment for other significant health-related issues. Additional services may include the outpatient traumatic brain injury clinic for evaluation or ongoing treatment for traumatic brain injury, the alcohol and drug dependency treatment program or its dual diagnosis affiliate, or referral for vocational rehabilitation services (evaluation or counseling).

Not all patients are appropriate for the integrated treatment approach utilized in the CPHE. Those with severe and disabling chronic pain or PTSD may require immediate referral to a more intensive specialized treatment program, such as the Trauma Recovery Program (TRP), which specializes in the treatment of PTSD, or the inpatient or outpatient Chronic Pain Rehabilitation Program. The TRP offers multiple evidence-based treatments for PTSD, including PE, Eye Movement Desensitization and Reprocessing (EMDR), and Cognitive Processing Therapy (CPT). Each of these treatment protocols offers exposure components to facilitate emotional processing and behavioral change. Minimizing the avoidance (i.e., cognitive, behavioral, and emotional) inherent in PTSD plays a critical role in each as well. The TRP also has available 10-week cognitive-behavioral treatment group for patients with mild comorbid chronic pain and PTSD who may not be in need of the more specialized/intensive pain care offered in the CPHE or CPRP. Participation in TRP programming does not preclude treatment in other services or clinics; however, the intensive specialized care provided maximizes opportunities for rapid stabilization and improvement. Once stabilization has occurred, concurrent treatment in programs that target accompanying comorbidities, such as chronic pain, may begin.

A similar pathway may be followed for those with severe and disabling chronic pain conditions where initial stabilization and treatment are provided within the CPRP, followed by referral to the CPHE, TRP, or other specialty care programs for subsequent treatment of associated comorbidities such as PTSD. The inpatient CPRP is an 19-day, intensive, interdisciplinary pain rehabilitation program that integrates CBT and behavior therapy with traditional rehabilitation therapies. The 8-week (twice weekly) outpatient CPRP provides the same comprehensive treatment as the inpatient CPRP, but care is provided on an outpatient basis in a less intensive fashion. Both programs incorporate the six treatment goals stated earlier in this section, and concurrent treatment of comorbidities such as PTSD occurs as needed.

Regardless of which programs are involved, these concurrent treatment efforts require close communication and collaboration between program staff in order to coordinate care, avoid duplication of effort, develop shared treatment goals, monitor progress, and optimize patient improvement. Within this service delivery framework, some indicated treatments may require more formal and direct resource sharing between programs or clinics. For example, one component of concurrent chronic pain and PTSD treatment at this southeastern VA is a joint CPHE/TRP psychoeducational outpatient group that provides education about the role of fear and avoidance in the maintenance of chronic pain and PTSD as well as the opportunity to increase readiness and motivation to pursue therapeutic interventions designed to systematically confront feared situations or activities common to both problem areas. A second 10-session conjoint group incorporates both psychological and active exercise treatments to address these comorbidities. Specifically, this group provides psychoeducation about the fear-avoidance model of chronic pain and PTSD, including a walking program (twice daily) and a hierarchy for PTSD-specific in vivo exposure assignments.

## 8. Conclusion

There are shared theoretical and practical factors in chronic pain and PTSD which stem from the avoidance and fear-based processes that typify both conditions. These can be conceptualized as a general failure to recover from acute physical or psychological injury. Given the shared mechanisms underlying each, we argue that in cases where the clinical presentation involves overlapping and interactive symptoms of both chronic pain and PTSD, concurrent treatment of shared components using evidence-based cognitive-behavioral interventions is a realistic and desirable treatment option. An interdisciplinary, outpatient program that provides integrated chronic pain and PTSD treatment has been developed and implemented within the VA stepped care model for pain management and rehabilitation to deliver this type of care. Outcomes data to date demonstrate broad-based physical, emotional, and functional gains by patients along with high levels of treatment satisfaction. We anticipate that this concurrent care approach will continue to develop and evolve as additional outcomes data and clinical experience become available. Nevertheless, confirmation of the extent and durability of these changes is needed.

Clinics and programs treating populations with comorbid chronic pain and PTSD should take into account the interaction between these conditions. Common factors may exacerbate the severity of both disorders and may prevent or delay adequate and adaptive recovery. However, these same commonalities may be advantageous from a treatment perspective in that some aspects of both chronic pain and PTSD are conducive to concurrent intervention. Confronting feared stimuli, which addresses the core fear avoidance issue characteristic of both chronic pain and PTSD, may be the single most effective way to prevent further functional decline or symptom escalation or to achieve significant and lasting symptom improvement. Further research is warranted to explore this clinical approach and its application across various clinical populations.

## Figures and Tables

**Table 1:** Summary of integrated care treatment components.

Treatment goal	Intervention
(i) Educate about the relationship between thoughts, emotions, and behaviors (ii) Increase awareness about the interaction between chronic pain, emotional functioning, and cognitive functioning (iii) Educate about the role of cognitions in emotional and behavioral functioning (iv) Educate about the interactions between chronic pain and PTSD (v) Educate about the role of fear-avoidance in the development and maintenance of chronic pain and PTSD	Psychoeducation

Reduce chronic pain and PTSD-related avoidance behaviors via systematic practice of increasingly challenging avoided stimuli	Construction of an in vivo hierarchy

Facilitate engagement in enjoyable activities to improve mood and challenge any inherent avoidances	Include behavioral activation activities on in vivo hierarchy

Correct attentional biases	Education; identification and cessation of safety behaviors

Normalize emotional experiences or responses and associated physiologic sensations	Normalization; education about fight or flight response; ongoing identification and systematic confrontation of avoidance or escape behaviors via real life exercises/activities

Reduce tension; regulate distressing physiologic sensations	Structured relaxation training to decrease stress yet preclude buffering from feared stimuli
